# A 10-year retrospective analysis (2012-2021) of hospitalizations resulting from dog bites in Southern Italy

**DOI:** 10.3389/fvets.2023.1104477

**Published:** 2023-02-21

**Authors:** Daniela Alberghina, Antonino Virga, Gianluca Sottile, Sergio Pio Buffa, Michele Panzera

**Affiliations:** ^1^Dipartimento di Scienze Veterinarie, Università degli Studi di Messina, Messina, Italy; ^2^Dipartimento di Scienze Agrarie, Alimentari e Forestali, Università degli Studi di Palermo, Palermo, Italy; ^3^Dipartimento di Scienze Economiche, Aziendali e Statistiche, Università degli Studi di Palermo, Palermo, Italy; ^4^Assessorato Regionale della Salute - Regione Sicilia, Palermo, Italy

**Keywords:** dog bite, hospitalizations, injury, Southern Italy, incidence

## Abstract

This study aimed to describe the incidence and characteristics of dog-bite injury hospitalizations (DBIH) in the largest administrative region of Italy (Sicily) over the 10-year period: 2012-2021. Four hundred and forty-nine cases were analyzed. Patients were divided into seven age groups: preschoolers (0–5 years), school-age children (6–12 years), teenagers (13–19 years), young adults (20–39 years), middle-aged adults (40–59 years), old adults (60–74 years), and the elderly (≥75 years). Association among categorical variables (age, gender, principal injury location) was evaluated using chi-square tests, and mean differences for normally distributed variables were assessed using one-way analysis of variance. Finally, a Poisson regression general linear model (GLM) analysis was used to model incidence data. The results revealed that the incidence of DBIH per 100,000 population increased from 0.648 in 2012 (95%CI 0.565–0.731) to 1.162 in 2021 (95%CI 1.078–1.247, *P* < 0.01). Incidence for both male and female victims also increased over the studied period (*P* < 0.05). We found an increasing trend of incidence in young and middle-aged adults (*P* < 0.05 and *P* < 0.005 respectively). Moreover, the most frequently injured age group by dogs was the preschooler group and, whilst we found a lower risk of being injured for males older than 20 years, no difference with females was observed. The location of lesions depended on the age group (*P* < 0.001). The number of days of DBIH increased significantly with age (*P* < 0.01). The increase of DBIH represents a public health problem that requires the development of preventive approaches.

## Introduction

Dog bites have been recognized worldwide as “a public health problem” that disproportionately affects children (1). Dog aggression also implicates animal welfare, with negative outcomes such as rehoming or euthanasia for the animals involved (2). While the number of pet dogs in Italy remained stable between 2014 and 2019, it then increased steadily in the following year: in 2020 the pet dog population in Italy amounted to ~8.3 million, an increase of around 18 per cent compared to the previous year (3). With the growth in the ownership of dogs in Italy, it is important to monitor the epidemiology of severe dog-bite injuries and their implications on public health. The number of European fatalities due to dog attacks between 1995 and 2016 increased significantly (4), but serious dog bites requiring hospital admission have also increased in the recent past (5, 6). This increase has been reported in Israel (7), in England (8), and in Poland (9). To date in Italy, only one preliminary attempt has been made to assess dog-bite related severe injuries requiring hospitalizations (10). In addition to physical impact, dog bites often carry psychological costs to the victim which are underestimated (11). This study has as its objective the provision of recent data regarding the incidence of dog-bite hospital admissions in the largest administrative region of Italy (Sicily) over the last 10 years (2012-2021) in order to progress toward better and more preventive measures to improve public safety.

## Methods

### Ethics statement

This study was approved by the Ethical Committee of the Department of Veterinary Sciences in Messina (No. 085/2022). All procedures were carried out in accordance with relevant Italian guidelines and regulations.

### Medical records extraction

The International Classification of Disease (ICD) is used to translate diagnoses of diseases and other health problems from words into an alphanumeric code, which permits easy storage, retrieval, and analysis of the data (12). We extrapolated from hospital discharge data, sent to the Public Health Department of the Sicilian Region, cases of dog-bite injury hospitalizations (DBIH) from 2012 to 2021 using the ICD-9, external cause of injury code E906.0 (dog bite). We used ICD-9 codes because in Italy they are still used within the context of hospital discharge. The Regional Health Service comprises of both Local Health Authorities and Hospital Authorities. Hospitals have the obligation to report cases of diseases to the Public Health Department of the Region which forwards them to the Ministry of Health. The latter coordinates the National Health Service. The detail regarding a bite is collected at the Emergency Room. It is here that after the clinical visit the patients can be discharged or hospitalized depending on the severity of the injuries. Data was anonymized and included only information about the patient's age, sex, lesion's location, date of hospital admission, and the number of hospitalization days. Patients were divided into seven age groups based on the stages of human development to best describe their epidemiological characteristics: preschoolers (0–5 years), school-age children (6–12 years), teenagers (13–19 years), young adults (20-39 years), middle-aged adults (40–59 years), old adults (60–74 years), and the elderly (≥75 years).

### Statistical analysis

We performed all statistical analyses using R statistical software version 4.1.1 (13). We used two-tailed *P*-values for all analyses, and the statistical significance was set at *P* < 0.05. Descriptive statistics (number, percentage, mean and SD) were used to summarize data. Differences between age groups year to year were assessed using a chi-squared test for trend in proportion. Association among categorical variables (age, gender, principal injury location) was evaluated using chi-square tests, and mean differences for normally distributed variables were assessed using one-way analysis of variance (ANOVA). Finally, a Poisson regression general linear model (GLM) analysis was used to calculate relative risk and confidence intervals for different age groups.

## Results

A total of 449 individuals were hospitalized and discharged during the studied period after dog-bite injury. The most frequently age group injured by dogs was the preschooler group (Table 1); all other age classes, except for the school-age group, who have a similar risk of preschoolers, showed significantly lower risk.

**Table 1:** Number of hospitalization cases and incidence per 100,000 inhabitants in Sicily between 2012 and 2021 per age class.

**Age group**	**Age class (years)**	**Number of cases**	**Incidence per 100,000 inhabitants**	**Relative risk**	***p*-value**
Preschooler	(0,5)	59	2.233	2.233 (1.730–2.882)	<0.001
School-age	(6,12)	66	1.934	0.866 (0.610–1.230)	0.422
Teenager	(12,19)	20	0.536	0.240 (0.144–0.398)	<0.001
Young adults	(20,39)	86	0.697	0.312 (0.244–0.435)	<0.001
Mild adults	(40,59)	121	0.842	0.377 (0.276–0.515)	<0.001
Old adults	(60,74)	68	0.825	0.369 (0.261–0.523)	<0.001
Elderly	(≥75)	29	0.581	0.260 (0.167–0.406)	<0.001

Statistical risk comparison was calculated using Poisson regression based on the Preschooler age class. We report 95% confidence intervals between brackets of the estimated relative risk.

**Figure 1:**
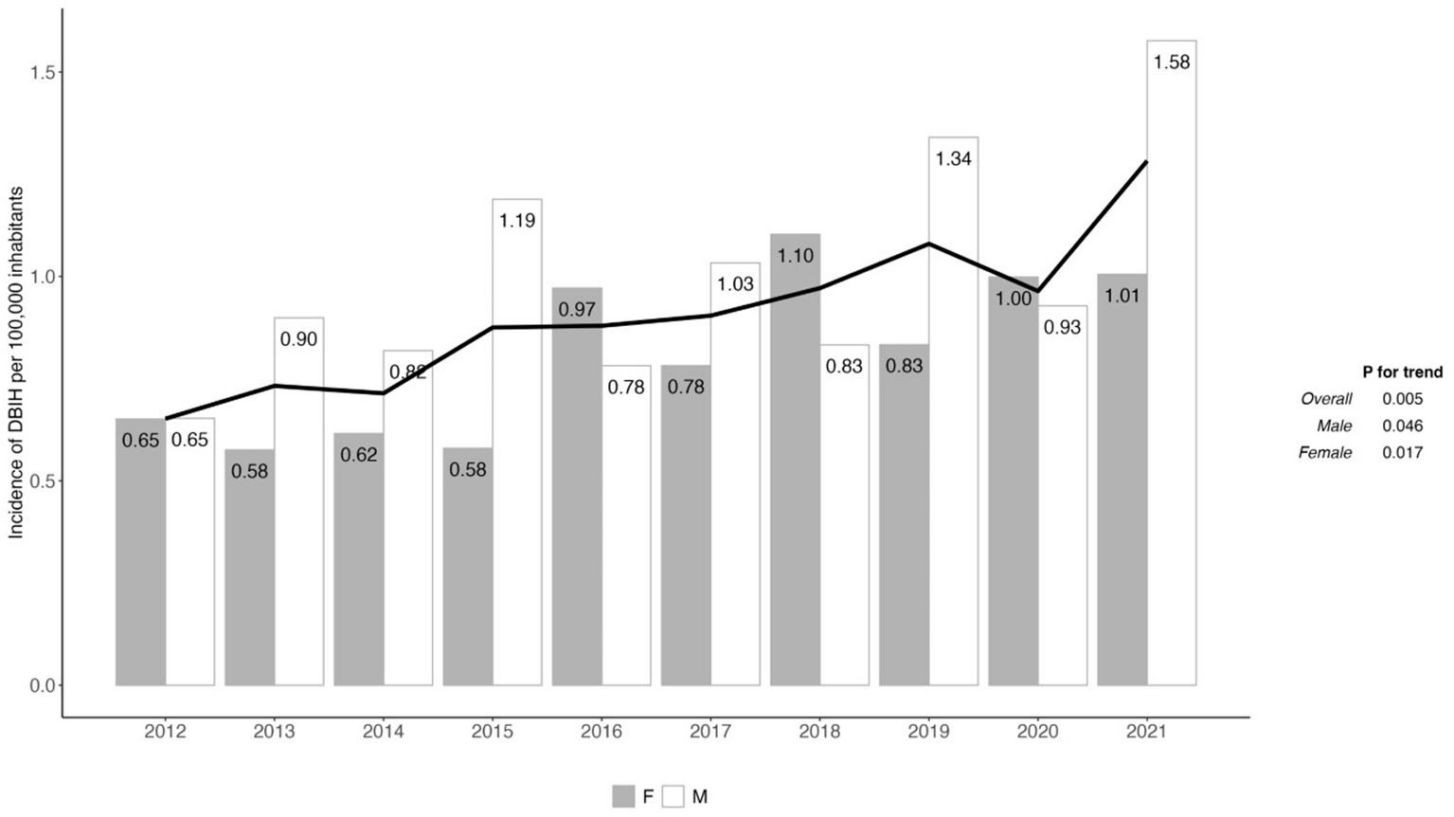
The trends of dog bite injury hospitalizations (DBIH) from 2012 to 2021 across both sexes. In particular, females showed an increase in the rates of DBIH over the 10 years examined.

As shown in Figure 1, the annual rate of dog-bite injury increased throughout the study period (*P* for trend < 0.01). In the sex-specific analysis, both males and females showed increases in the rate of dog-bite injury hospitalizations (*P* for trend < 0.05).

The total numbers of pre-schoolers and school-age victims (*n* = 125) is similar to the number of middle-aged adults (*n* = 121). Therefore, despite a similar number of bites per age group, the different age ranges mean that children (age 0–12) received proportionally more bite injuries than middle-aged adults (age 40–59).

**Figure 2:**
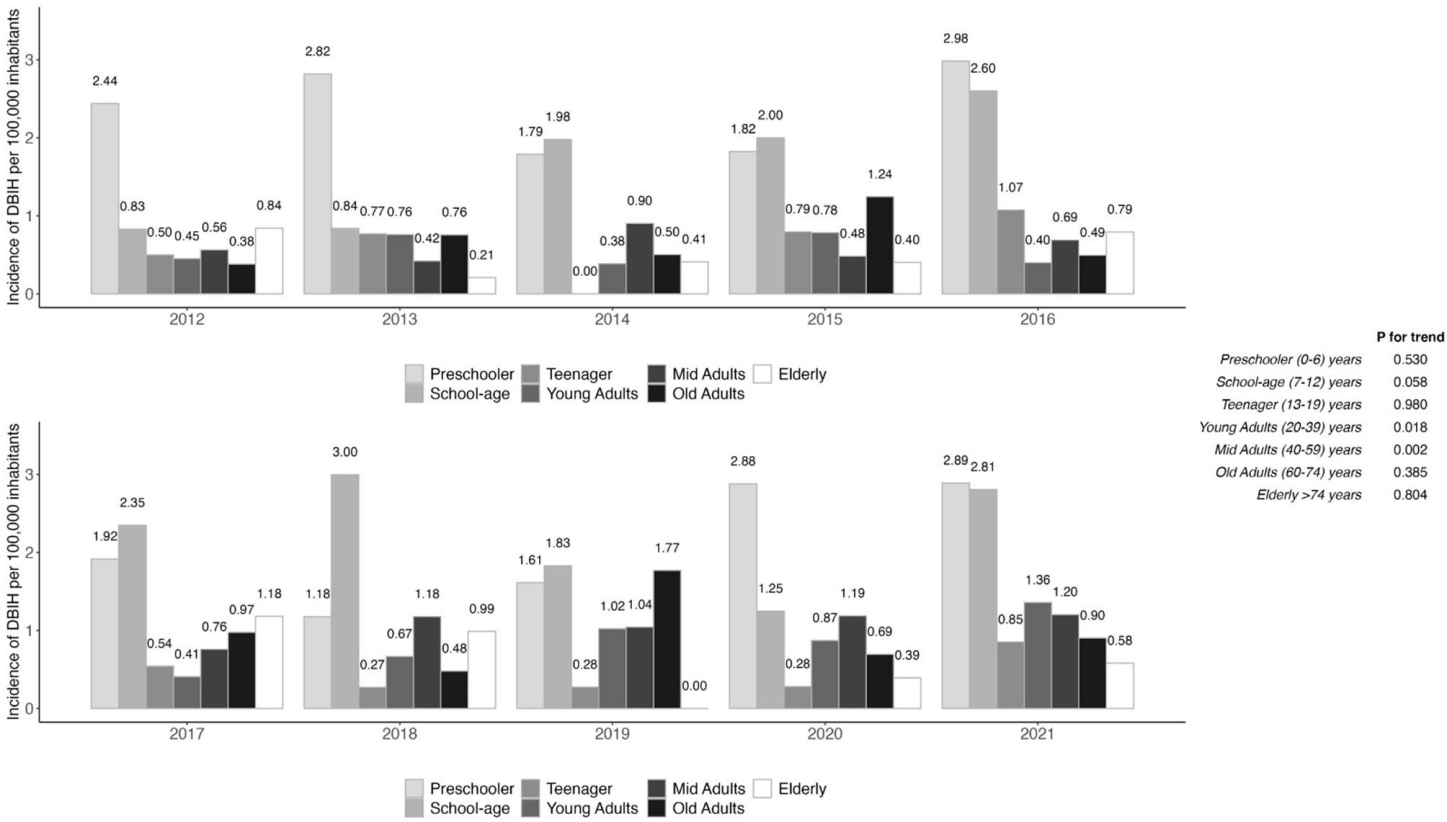
Evaluation of trends indicates an increase in young (20–39 years) and middle-aged adults (40–59 years).

The mean age of each age group and the sex of patients are reported in Table 2. Except for teenage and middle-aged groups, the percentage of males was higher than that of females in all groups. The middle-aged group was the only group where female victims were more numerous than male victims. The risk associated with males was significantly higher in the “male preschool age group” and the “school-age group” in comparison to all other groups (*P* < 0.001). No significant difference was observed for females in all groups. The association among the variables age and sex was not significant (*P* = 0.34). Figure 2 shows trends in the age-specific groups of DBIH between 2012 and 2021. An increase in rates was observed in young and middle-aged adults (*P* for trend < 0.05), but there were no significant increasing trends for the other age groups. A significant relationship was found between injury location and age (*P* < 0.001, Table 2). The risk of receiving a dog bite injury to the neck, face and head rather than to another body part than in the other age groups. The risk of receiving a dog bite on the arms or hands in patients over the of 20 years and in the lower extremity in patients over the age of 40 years was significantly higher than in other age group. The mean number of days of DBIH increases with age (*P* < 0.001, Table 2).

**Table 2:** Distribution of dog bite injuries hospitalizations (DBIH) and incidence per 100,000 inhabitants in Sicily between 2012–2021 according to age and sex of patients, principal lesion location and number of days of hospitalizations.

	**Preschooler (*N* = 59)**	**School-age (*N* = 66)**	**Teenager (*N* = 20)**	**Young adults (*N* = 86)**	**Mid adults (*N* = 121)**	**Old adults (*N* = 68)**	**Elderly (*N* = 29)**
Age group (years)	(0,5)	(6,12)	(13,19)	(20,39)	(40,59)	(60,74)	(75,90)
Mean age (± S.D)	3.93 (1.62)	9.15 (1.61)	16.4 (2.35)	29.7 (5.62)	49.7 (5.65)	65.9 (4.59)	79.3 (3.43)
Percentage	13.1%	14.7%	4.5%	19.2%	27.0%	15.1%	6.5%
**Gender:** ***P*** **value** = **0.340**							
Male	**34 (1.287)**	35 (1.025)	10 (0.268)^***^	47 (0.381)^***^	58 (0.404)^***^	40 (0.485)^***^	18 (0.361)^***^
Female	25 (0.946)	31 (0.908)	10 (0.268)	39 (0.316)	63 (0.438)	28 (0.340)	11 (0.220)
**Injured body part** ***P*** **value**<**0.001**							
Neck, face, head	**44 (1.665)**	41 (1.201)	10 (0.268)^***^	26 (0.211)^***^	28 (0.195)^***^	9 (0.109)^***^	2 (0.04)^***^
Arm	3 (0.114)^***^	9 (0.264)	3 (0.08)	22 (0.178)^**^	28 (0.195)^***^	16 (0.194)^***^	15 (0.301)^***^
Hand	2 (0.076)^***^	2 (0.059)	7 (0.187)^**^	30 (0.243)^**^	44 (0.306)^***^	29 (0.352)^***^	7 (0.14)^***^
Trunk	0 (0.000)	1 (0.029)	0 (0)	1 (0.008)	0 (0)	1 (0.012)	0 (0)
Lower extremity	9 (0.341)^***^	11 (0.322)	0 (0) [–]	5 (0.041)	20 (0.139)**	13 (0.158)^***^	5 (0.1)^**^
Not specified	1 (0.038)^***^	2 (0.059)	0 (0)	2 (0.016)	1 (0.007)	0 (0)	0 (0)
**Number of days (mean** ±**SD)** ***P*** **value**<**0.001**						
	4.19 ± 3.8	4.73 ± 3.8	3.93 ± 2.7	6.07 ± 5.2	6.80 ± 5.8	7.87 ± 8.1	10.4 ± 8.5

Significance was calculated by Poisson regression. The variables, Preschooler's age group male and age group lesions to the neck, face and head, in bold, were fixed as the reference category for statistical analysis. The p-values in rows are referred to an ANOVA test. Asterisks in columns denote statistical differences with respect to the reference category: ^***^ < 0.001, ^**^ < 0.01, ^*^ < 0.05.

## Discussion

From the results of this study, we can presume that dog-bite injuries in Southern Italy are growing as a public health problem. This work has identified an increase in hospital admissions in Sicily due to dog bites and a doubling of this incidence over 10 years. The trend showed a significant increase in cases, both for males and females. In this study, the victims most frequently injured were children between the ages of 0 and 5, a figure in keeping with previous studies in Italy (10, 14, 15) and worldwide (16–19). The proportion of number of cases between preschoolers and schoolers was almost 1 (0.89). In a study in the USA and Korea, the highest proportion of dog-bite injuries was reported in school-aged children aged 7–12 years (20, 21). In a recent review, it is reported that children under the age of 2 years and between the age of 9 and 12 years are most bitten (22). Children may be more prone to dog bites because of miscommunication in body language between dog and child (23). A previous study reported a decrease in bite rates for children in the USA (24).

Evaluation of trends also indicates an increase in young (20–39 years) and middle-aged adults (40–59 years). The latter was the only age group where female victims were more numerous than male victims. This result agrees with a recent study in the UK (8) and authors hypothesized that any behavior or interactions that make predominately middle-aged women more susceptible to being bitten and admitted to hospital should be explored. From a questionnaire administered to the Italian public, women are more willing to spend time with their dog than men (25) and this could help to explain this trend. Previous studies found an association between gender and patients injured by dogs, with more males being injured than females (14, 26). To the authors' knowledge, few papers describing hospitalization mention proportional increase of adult dog-bites admissions among admission in general (8, 19, 21). The reasons for this finding are unknown and further research is required to understand the causes of these data patterns. This absence of an increasing trend of child admission, may be caused by the reduced number of children playing outdoors in the modern era (21). The percentage of lesions to the face, head, and neck decreases with age. Seventy-five percent of bites in preschooler children are distributed in this area, a finding that has been previously reported in other studies (17, 26–28). This finding could be explained by the height of a 3–5 years old child, which could be similar to that of a standing medium-large dog (29). It is also possible that these areas are the most affected parts in children because of how they interact with dogs. At this age, a child could behave in a way perceived as a challenge to a dog, even if the dog is not typically aggressive (30). Furthermore, even in the context of the play, a bite to a 3–5-year-old may require hospitalization. Preschool children cannot recognize a dog's emotions and behave appropriately around dogs (31). Younger children are less good at interpreting dog behavior, and they are particularly poor at recognizing fear in dogs (32). Fearful dogs may be more likely to show aggressive behavior toward children (22). Moreover, dogs could lack previous experience with children: a recent study showed that dogs that had been in contact with children during their socialization period did not show aggressive behavior or excited behavior toward the child in behavioral test (33). It is interesting to mention that there is also variability regarding the breakdown of injuries among adult groups: in young, middle-aged, and old victims, arms and hands were the most injured areas, while in the older people, the lower extremity was the most lesioned. This difference could be related to a variability of interaction among adults and elderly. This result agrees with a previous study where the most common lesion from dog bites in adults is on the hands (34). Another explanation could be the form of interaction: a recent study found that if the upper extremities were bitten, it was likely the person approached the dog, whereas for the lower extremities, it was more likely the dog approached the person (35). Unfortunately, we have no information on this subject.

The way in which age affects the number of hospitalization days could be due to the extra protection taken for children, while for elderly the increase of the length of DBIH could be as a result of independent medical conditions prolongs their recovery (34).

Further studies are warranted to determine the mechanism of dog attacks on humans across different age groups. These studies will help determine whether dog bites are associated with human characteristics and the extent to which these characteristics foster aggression. Educational programs for owners are fundamental tools to reduce aggression risk factors and prevent aggression (36). Some limitations must be acknowledged in the present study. The study is focused on hospitalization data and children are more likely to be found in hospital records than adults. We were unable to collect and analyze the circumstances of the dog bites, e.g., familiarity or not between the victim and dog. This limited the interpretation of some findings. It is necessary that in the case of DBIH, the veterinary services are promptly alerted to obtain more information on the specifics of the injury. Unfortunately, this almost never occurs in Southern Italy. The emergency room doctor must report the dog bite at the Local Health Authorities. During the drafting of this paper, the authors contacted the Veterinary services of Local Health Authorities, but it was not possible to trace the information because there were almost no reports corresponding to our data. The Authors hope that information flows related to DBIH between doctors and veterinary services will be better regulated. Another limitation of the study is that less severe injuries not requiring hospitalization were not included. The true incidence of dog bites is likely to be underestimated (35). Research is required to develop new effective intervention strategies in response to biting victims' changing demographics to minimize the risks of living and working with dogs.

## Conclusion

Data related to hospitalization admissions have unique opportunity to give information about severe injuries from dog bites. They are, however, only the tip of the iceberg of this problem. Collaboration with veterinary services is essential to develop prevention strategies.

## Data availability statement

The original contributions presented in the study are included in the article, further inquiries can be directed to the corresponding author.

## Ethics statement

The studies involving human participants were reviewed and approved by Ethical Committee of the Department of Veterinary Sciences in Messina (No. 085/2022). The Ethics Committee waived the requirement of written informed consent for participation.

## Author contributions

Methodology and writing—original draft preparation: DA. Analysis: GS. Resources: AV and SPB. Data curation: MP and DA. Review and editing: MP. All authors have read and agreed to the published version of the manuscript. All authors contributed to the article and approved the submitted version.
